# Socio-Environmental Vulnerability Index: An Application to Rio de Janeiro-Brazil

**DOI:** 10.3389/ijph.2021.584308

**Published:** 2021-03-29

**Authors:** Fernanda Siqueira Malta, Eduarda Marques da Costa

**Affiliations:** ^1^ Instituto Brasileiro de Geografia e Estatística, Rio de Janeiro, Brazil; ^2^ Center of Geographical Studies, Institute of Geography and Spatial Planning, Universidade de Lisboa, Lisbon, Portugal

**Keywords:** vulnerability, socio-environmental vulnerability index, geographic information systems, decision support systems, Rio de Janeiro

## Abstract

**Objectives:** The concept of vulnerability has been used more frequently in several studies, in an attempt to better understand the specificities and needs of different population groups, both in environmental and socio-economical terms. The aim of this study is to identify, characterize and analyze populations in situations of socio-environmental vulnerability in the city of Rio de Janeiro, based on social, economic, environmental and public health indicators organized into a summary index – the Socio-Environmental Vulnerability Index.

**Methods:** The methodology integrated 15 indicators in a Multi-Criteria Decision Analysis into a Geographic Information System.

**Results:** According to our results, socio-environmental vulnerability in Rio de Janeiro is aggravated by at-risk situations and environmental degradation. These aspects are jeopardized by the population density in slum areas, where the most disadvantaged groups live in a process of environmental and urban exclusion.

**Conclusion:** The study concludes about the importance of these tools in guiding resource allocation and their contribution to formulating and implementing more effective public policies.

## Introduction

In recent years, the term vulnerability has become a keyword in studies on environmental risks and climate change issues. At the same time – and this may be one of the reasons for its growing popularity – vulnerability is a rather diffuse term [1]. Various definitions of vulnerability have emerged and are used in different disciplinary contexts, whether related to sustainability [2], natural and environmental risks [3, 4] within the context of climate change [5, 6] or in social and economic fields [7–10]. Another approach to vulnerability that is growing relevance, can be seen in the context of health [11, 12]. “The concept of vulnerability implies some risk combined with the level of social and economic liability, and the ability to cope with the resulting event. Thus people become “vulnerable” if access to resources either at a household, or at an individual level is the most critical factor in achieving a secure livelihood or recovering effectively from a disaster” ([13], pp. 370, 371).

In epistemological terms, Hufschmidt [14] classified vulnerability in two research areas: “the ‘human ecologist school’, also labelled the ‘Chicago school’ [15] or ‘behavioral paradigm’ [16, 17], and the ‘structural paradigm’ (or better ‘view’) associated with Sen’s [18] ‘entitlement’ approach” (p.623).

The concept of vulnerability become more commum and broad, progressively adapted for each field of knowledge [19]. Another perspective [20], considered that in social and economical context, vulnerability follows three approachs: “economic strengthening, poverty and social exclusion”; “multiple pathways of vulnerability” (initially pointed by [21], p. 268); and the “resilience perspective”.

For the first approach [22], authors conceptualize vulnerability in terms of either poverty dynamics, food security or sustainable livelihoods. However [23], quoting [24, 25], consider that vulnerability due to poverty, is not only dependent on current income inadequacy, but rather insecurity and exposure to the risk of future low income. Also other authors consider that poverty cannot be conflated with vulnerability, and that vulnerability analysis in the scope of poverty, requires forward-looking information including indicators of risk [20, 26–28].

In that context, the second perspective of Adger [21], points to a more integrative and systemic approach, assuming that vulnerability results from various causes and effects of vulnerability, integrating natural hazards, social vulnerability, and economic vulnerability.

In the last years, the third perspective emerges in the literature. The discussion of vulnerability linked to resilience process emerged due to evidence on the capacity of some communities to face with external pressures resulting from social, political and environmental change [29], showing the capacity for adaptative action [21]. This adaptative actions are supported by the development of new vulnerability tools and methods across resource management, social change, urbanization and climate change stress factors [5, 7, 21, 30–34].

The literature defends that level of vulnerability depends on the balance between risk and responses of the system. Ones [35] presents the notion of ‘spatial vulnerability’ as the “Access to a fair distribution of resources within social space shapes and is shaped by the nature and degree of vulnerability” (p. 230), while for Cutter et al [7] the concept of social vulnerabilty is related to: gender, race and ethnicity, age, family structure, employment, poverty, limited access to resources such as information, knowledge and technology, limited access to political power and representation (marginalization, exclusive), limited social capital including social networks and connections, vulnerable housing and low access to critical services such as communication, transportation, power supply, water supply, sanitation, education, medical services and other.

Another definition of social vulnerability considers four dimensions: socioeconomic status, household composition and disability, minority status and language and infrastrucutres and services [36, 37] while other [38], discuss the perspective of social equity in the definition of urban resilience planning based on distributional, recognitional, and procedural equity dimensions.

These dimensions are represented by indicators that could be organized depending the objective of the analysis: context-oriented, outcome-oriented and participation/actor-oriented perspectives [39]. Measures of vulnerability can not be represented by a single indicator defending the need to create index or typologies supported in multiple indicators related to distinct dimensions of vulnerability and, in particular, social vulnerability.

The construction of the index is based on the production of indicators. These can be defined as a measure, most often quantitative, used to replace, quantify or operationalize a concept [40]. Indicators are considered, more and more, effective tools used to support planning and policy making activities, the resource allocation and the definition of priorities in the different spheres of government. Indicators enable, for example, the monitoring of the population’s living conditions as well as the economic situation of a country ([41, 42]) and in that context a good indicator should be sensitive to the changing conditions of the environment and of society, be specific to the problem under analysis, be reproducible according to established methodological standards, provide a prompt response, be understood by the population, be robust to changes in methodology and be easily available or of low-cost [43].

The construction of indicators to assess living conditions and monitor public policies gained momentum in the 1990s with the United Nations introduction of the Human Development Index (HDI), devised by economist Mahbub ul Haq with the collaboration of economist Amartya Sen, winner of the 1998 Nobel Prize in Economics [44]. The HDI is a summarized measure of long-term progress in three basic dimensions of human development: income, education and health. This index has had major repercussions worldwide mainly because it is simple and easy to understand and represents the most holistic and comprehensive way of measuring development. Although the HDI broadens the perspective on human development, it does not cover or exhaust all aspects of development, including the issue of vulnerability. Therefore, new indices were developed to measure a diversity of themes not covered by the HDI.

The UNICEF’s Multiple Indicator Cluster Survey (MICS) data is relevant to vulnerability as understood through the lens of “health, education, child protection and HIV/AIDS” [45]. Another useful source of secondary data is the World Bank’s Living Standards Measurement Survey (LSMS), which includes survey data at community and household levels, including information on pricing and consumption to provide information on living standards [10]. The third example is the Social Vulnerability Index (SoVIVR) created at the University of South Carolina [7] and the identically named Social Vulnerability Index (SVI) developed at the U.S. Centers for disease Control [46].

In Brazil, in the 1990s and 2000s, several actions were taken to create indices that could portray the socio-economic reality of different population groups. These included the Social Exclusion/Inclusion Index [47], the Family Development Index - IDF [48], the Youth Vulnerability Index - IVJ [49] and the Urban Quality of Life Index - IQVU [50].

From 2010 on, other indices were developed to substantiate the development of public policies specifically targeted at population groups considered to be more vulnerable. Some examples are the São Paulo Social Vulnerability Index of the *Fundação Sistema Estadual de Análize de Dados de São Paulo* [49], the Health Vulnerability Index of the Municipality of Belo Horizonte, the Social Vulnerability Index of the Institute of Applied Economics Research and the Municipal Vulnerability Index of the Oswaldo Cruz Foundation [6].

Although the use of an accepted index as a benchmark to demonstrate the operationalization of social vulnerability, the index alone is insufficient to demonstrate model validity [51, 52]. For the authors, other methodologies could be reported to measure vulnerability, like cluster analysis involving diverse type of indicators, as presented for the Micro-regions and Meso-regions in Minas Gerais-Brazil [53].

The issue of vulnerability is complex and for each situation, vulnerable population and region needs specific information; for this reason, there are several indices, each one developed for a given reality, with different objectives and uses. In this context, this study proposes the creation of an index to analyze the “socio-environmental vulnerability”, integrating social, economic and urban infrastructure processes related to the precarious living conditions of the population (work, education, income, sanitation, mobility) with environmental, health and public safety conditions. The index is presented in a context-oriented perspective and their update could contribute to support the formulation and implementation of public policies, since for these actions it is essential to spatially locate the areas where the most vulnerable population are concentrated.

### The Studied Area: Rio de Janeiro

In addition to having the highest concentration of population in the state, Rio de Janeiro is a metropolis that has long been known for its social inequality as well as problems regarding urban infrastructure, environmental risks and deficiencies in the health system and public safety [54–56]. This reality justifies the study of social and environmental vulnerability in the municipality, with the purpose of guiding the creation of public policies and the allocation of more appropriate public resources based on scientific evidence, resulting from a diagnosis made with information suited to the territory, scale and adequate time period.

Rio de Janeiro is the Brazilian city with the largest contingents of people living in subnormal housinf clusters, a technical name used by the Brazilian Institute of Geography and Statistics (Instituto Brasileiro de Geografia e Estatística - IBGE [57] to designate locations with informal housing built from fragile materials, invasions and a minimum of 51 households. Another key criterion for classifying such areas as subnormal housing clusters is the lack or inadequacy of basic public services such as water supply, sewage and rubbish collection services, in addition to generally being locations that are laid out in a dense and disorderly manner.

**FIGURE 1:**
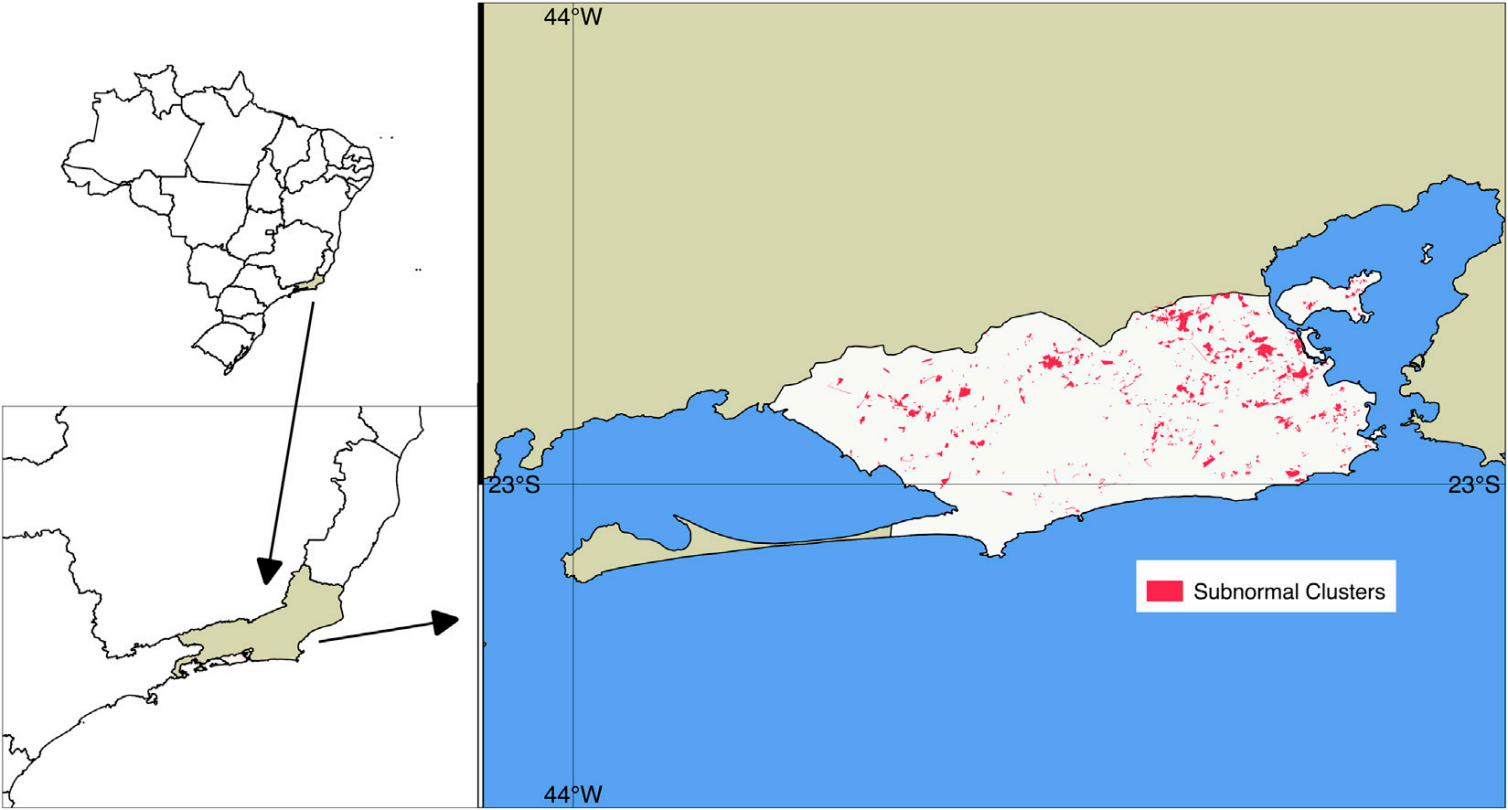
Subnormal housing clusters in the municipality of Rio de Janeiro in 2010. Source: Instituto Brasileiro de Geografia e Estatística 2010.

According to the 2010 Demographic Census, 23% of the population of Rio de Janeiro live in subnormal housing clusters, commonly known as *favelas*. The proportion of people living in these locations varies significantly in the municipality, with great predominance in the central region, where although in absolute terms the population living in *favelas* is the smallest in the municipality, its proportion in relation to the total population is the largest, representing 35% of the inhabitants of the region. Next is the northern region of the municipality with 27% of its population living in *favelas*, then Barra da Tijuca and Jacarepaguá region with 26%, the southern zone with 17% and finally the western zone with 16%. Figure 1 shows the spread of the 763 subnormal clusters in the city of Rio de Janeiro.

The existence of this number of subnormal housing agglomerates points to a serious problem related to an inconsistency between the municipality’s housing policies and the demand for housing. Population growth in these regions between 2000 and 2010 was 27.7%, while the regular city, with the exception of *favela* dwellers, grew at a rate that was eight times lower - only 3.4%.

The presence of these *favelas* is a tragic indicator of economic dynamics and reveals the result of a lack of effective social policies over recent decades. In the city of Rio de Janeiro, hundreds of *favelas*, most of them on slopes or on river banks, create risky living conditions due to landslides and flooding [58] and the increasing climate change impacts in the territory [59, 60].

The amplitude of these urban problems and their impact on different territorial scales justify an interest in understanding and analyzing the issue of people living in situations of vulnerability in Brazil and particularly in the Rio de Janeiro municipality, where the problem of the *favelas* has become chronic.

The study area covers the entire municipality of Rio de Janeiro, the capital of the state of Rio de Janeiro, located in the south-eastern region of Brazil. Rio de Janeiro has an area of 1,200 km^2^ where approximately 6.3 million people live, according to the 2010 Census, amounting to 40% of the state’s total population. The Rio de Janeiro municipality is a fully urbanized region and is divided into Planning Areas (PAs), a division used by the city council to administratively serve the entire municipality.

**FIGURE 2:**
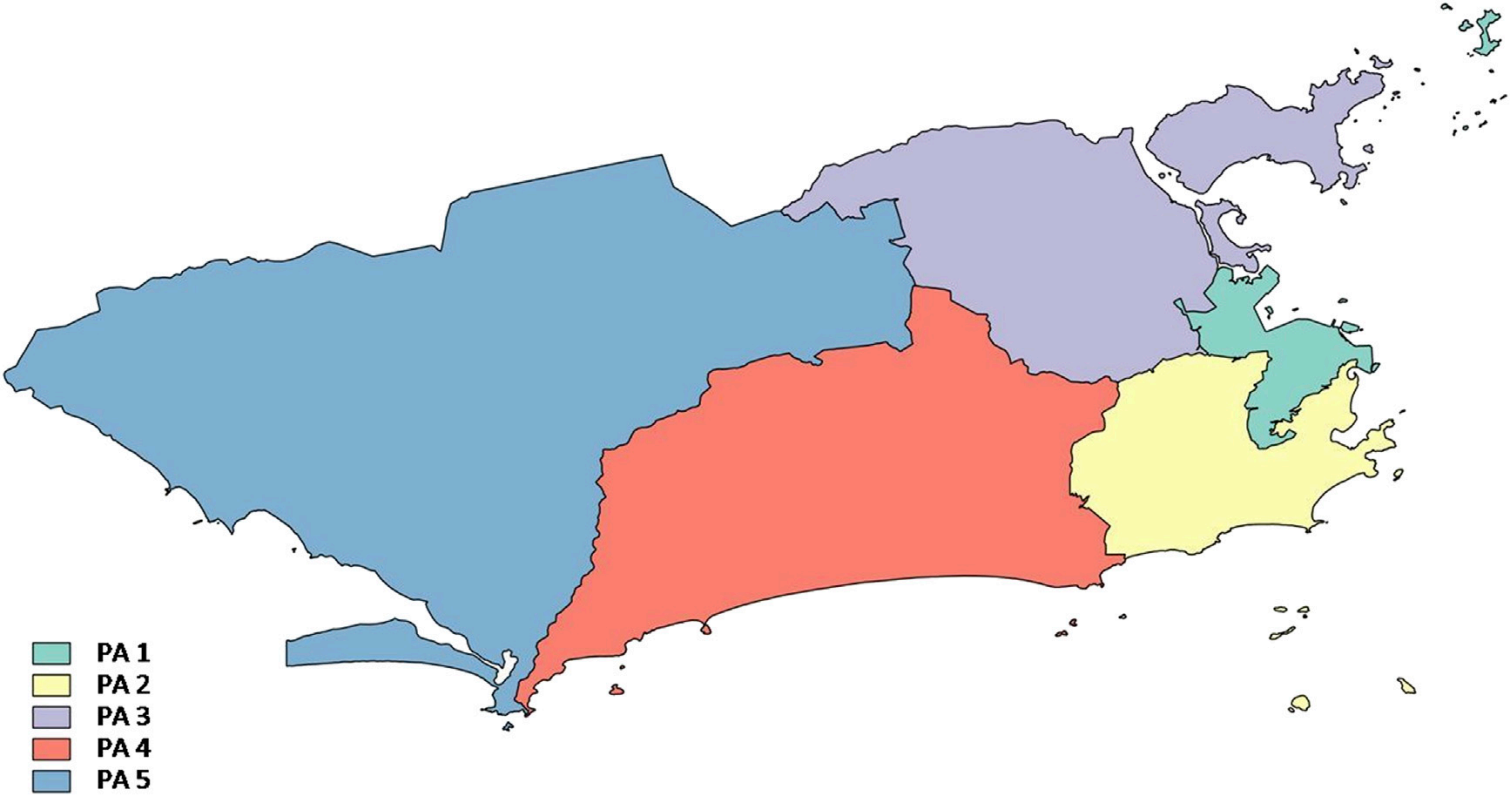
Municipality of Rio de Janeiro by Planning Area. Source: Prepared by the authors from the city council of Rio de Janeiro, 2010.

Planning area 1 (PA 1) is the region of the historic center of the city, and also the area that has undergone the largest transformation in the urban scenario. Planning area 2 (PA 2), known as the South Zone, corresponds to the expansion area of the city promoted by the implementation of a tramway system in the second half of the 19th century, and is located between the Atlantic Ocean and the Tijuca Massif. Planning area 3 (PA 3), also known as the North Zone, concentrates the largest population contingent in the municipality (40%) as it is the most densely populated. Planning area 4 (PA 4) has an extensive lowland area bounded by the Tijuca and Pedra Branca massifs and the Atlantic Ocean. This region encompasses the neighbourhoods of Barra da Tijuca and Jacarepaguá. Planning area 5 (PA 5), also known as the West Zone, has the second largest population of the municipality and the lowest population density. The low density is due to the fact that this is a region with a vast territory. This region includes the three most populous neighbourhoods of the city: Campo Grande, Bangu and Santa Cruz (Figure 2).

## Methods

The Socio-Environmental Vulnerability Index (SEVI) was built by integrating multi-criteria decision analysis (MCDA) methodology, more specifically the analytic hierarchy process (AHP), with a Geographic Information System (GIS) [61]. The proposed SEVI is made up of fifteen indicators based on bibliographic review and discussions with specialists in the fields of Sociology, Economics, Urban Infrastructure, and Environment, Health and Safety.

These indicators were grouped into three components: Socio-economic, Urban Infrastructure, and Environment, Health and Safety. Each component is made up of the indicators below.

The indicators used in the construction of the SEVI seek to highlight different situations that indicate exclusion and vulnerability in the Brazilian territory, in a perspective that goes beyond identifying poverty perceived merely as insufficient monetary resources Table 1. The components of the SEVI correspond to sets of assets, resources or structures whose access, absence or insufficiency indicate that the standard of living is low, suggesting, at the limit, non-access and non-observance of social rights.

**Table:** 

0.000 – 0.200	Very low
0.201 – 0.300	Low
0.301 – 0.400	Medium
0.401 – 0.500	High
0.501 – 1.000	Very high

**TABLE 1:** List of Indicators that support the Social-Environmental Vulnerability Index (SEVI).

*Components*	Indicators	Sources
*Socio-economic Component*	Indicator 1: Percentage of mothers who are head of the household, who did not complete basic education and who have at least one child under the age of 15	Census, 2010 [57]
	Indicator 2: Percentage of children living in households where none of the residents completed basic education	Census, 2010 [57]
	Indicator 3: Percentage of people aged 15 to 24 who do not study, do not work and whose per capita household income is equal to or less than half the minimum wage	Census, 2010 [57]
	Indicator 4: Proportion of people whose per capita household income is equal to or less than half the minimum wage	Census, 2010 [57]
	Indicator 5: Percentage of people aged 18 or over who did not complete basic education and are in an informal occupation	Census, 2010 [57]
*Urban Infrastructure Component*	Indicator 6: Percentage of people living in households with a per capita income of less than half the minimum wage and commuting over 1 h to get to work	Census, 2010 [57]
	Indicator 7: Ratio of residents per household	Census, 2010 [57]
	Indicator 8: Percentage of households without a storm drain/manhole – openings that give access to underground enclosures through which rainwater drains	Census, 2010 [57]
	Indicator 9: Percentage of people in households with inadequate water supply and sewage	Census, 2010 [57]
	Indicator 10: Percentage of people in households with no rubbish collection service	Census, 2010 [57]
*Environment, Health and Safety Component*	Indicator 11: Susceptibility to slippage	Fundação geo-rio 2013 [62]
	Indicator 12: Risk of inundation and flooding (source: Index of susceptibility of the environment to flooding - 2010)	Miranda 2016 [63]
	Indicator 13: Mortality up to one year of age	Census, 2010 [57]
	Indicator 14: Percentage of households without trees in their yard	Census, 2010 [57]
	Indicator 15: Violent lethality	Instituto de segurança pública – Rio de Janeiro 2013 ([64])

Source: Selected by the authors.

After selecting the above-mentioned indicators, the proposed methodology is divided into three stages. Data entry is one of the major stages and requires special care. The indicators used in the construction of the SEVI were standardized, becoming dimensionless and varying between 0 and 1, where the vulnerability is greater the closer it gets to 1. It should be noted that zero does not represent the absence of vulnerability but the smallest relative value, and vice-versa for a value of 1.

After this stage, the standardized indicators were transferred to a GIS and the multi-criteria analysis methodology for SEVI construction was applied to them.

Due to methodological choice and for analytical simplicity, the Analytic Hierarchy Process (AHP) method was used. It was created in the 1970’s by Thomas L. Saaty [65] and is one of the main methods of the American school. This method is based on the hierarchical structure of a complex problem, suited to the analysis proposed by our study, which is related to socio-environmental vulnerability. This structure consists in defining the global objective and decomposing the system into several levels of hierarchy, enabling us to see the system as a whole as well as its components. In addition, it is possible to analyze the interactions of the components of the decomposition and to ascertain the impacts that they exert on the system.

**FIGURE 3:**
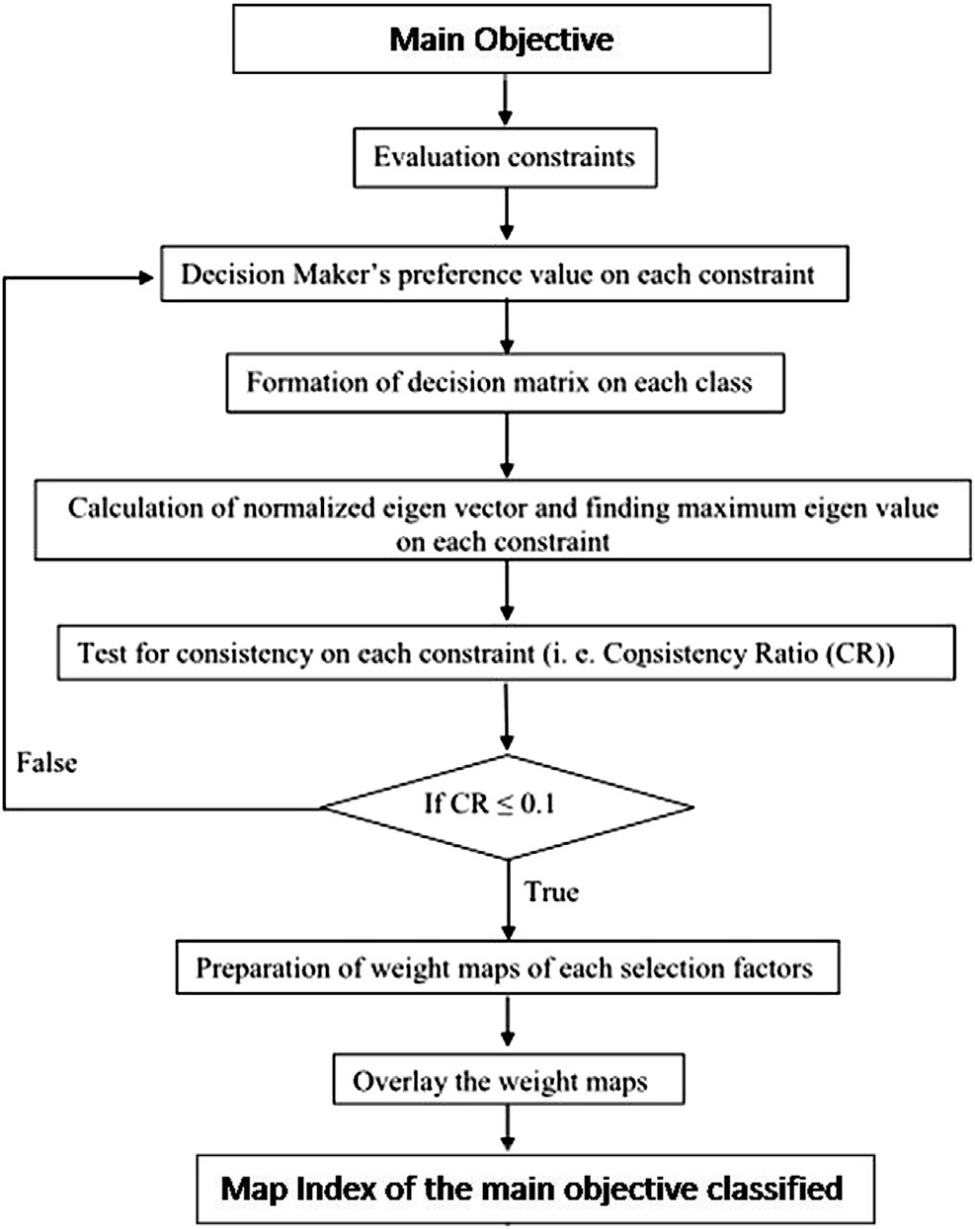
Conceptual flowchart of the AHP. Source: Prepared by the authors.

The AHP enables a structured decision-making approach using the judgment of specialists, and it includes five stages: 1) definition of the problem, 2) hierarchical construction and development of the problem into component factors related to the objectives of the problem, 3) construction of the comparison matrix, 4) calculation of the Eigen vector, main Eigen number, consistency index and consistency ratio, and 5) if there are inconsistencies in the decision process, revision of the comparison matrix until a consensus is reached. The conceptual flowchart of the AHP is shown in Figure 3.

**FIGURE 4:**
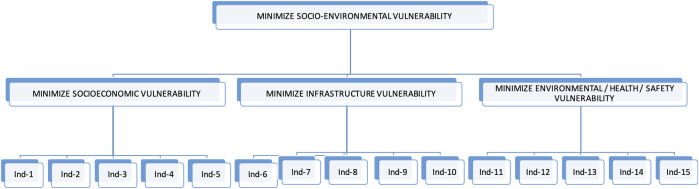
Hierarchy of objectives of the multi-criteria analysis. Source: Prepared by the authors.

Starting from the construction of the hierarchy, the criteria, i.e., the 15 indicators of the SEVI’s three components Figure 4 were compared on a pair-to-pair basis according to their importance in achieving the main objective – minimizing Socio-environmental Vulnerability. This comparison was substantiated by the relative importance scale [65], based on the analysis of specialists in the fields of Sociology, Economics, Urban Planning, Environment, Health and Safety.

After normalizing the original comparison matrix, it was possible to calculate the weight of each indicator (Eigen vector) and the main Eigen number (*λ*_max_) $\left(\lambda_{max}\right)\;$ which is obtained through the sum of the product of each element of the Eigen vector by the total of the respective column of the original comparison matrix).

Having obtained the (*λ*_max_) $\left(\lambda_{max}\right)$ , it was possible to calculate the value of the Consistency Index (CI) of the comparison matrix using the formula:

$$\text{CI}=\frac{\left(\lambda_{max}-n\right)}{\left(n-1\right)}$$

where *n* is the matrix order, which in this case equals 15.

Saaty [65] proposes the calculation of the Consistency Ratio (CR), obtained with the formula:

$$\text{CR}=\frac{\text{CI}}{\text{RCI}}\text{,}$$

where CI corresponds to the Consistency Index and RCI corresponds to the Random Consistency Index calculated for square matrices of order n by the Oak Ridge National Laboratory in the United States. If CR is greater than 0.1, the comparison matrix is inconsistent and should be revised. Since CR < 0.1 the comparison matrix is consistent. For *n* = 15, RIC = 1.59 and we obtain the value of CR = 0.0997.

After this stage it is possible to construct the normalized maps for each indicator-criterion, apply the AHP to the criteria maps and finally obtain the index map of the main classified objective.

Statistical processing of the data was carried out by means of SAS software and the mapping, spatial analyses and AHP by means of the QGIS open source program.

## Results

As mentioned above, the SEVI ranges between 0 and 1, and the closer to one it is, the greater the socio-environmental vulnerability; conversely, the closer to 0 it goes, the lower the vulnerability. For better applicability, this index was divided into five equal bands:

The results were presented by PA, in accordance with the territorial macroplanning approach existing in the city council of Rio de Janeiro.

Approximately half of PA 1’s area is classified as having medium socio-environmental vulnerability (48.3%), 27.4% as having low socio-environmental vulnerability, 23.6% as having high socio-environmental vulnerability and 0.7% as having very high socio-environmental vulnerability. The highest point of the region's very high vulnerability lies in the Morro da Providência *favela*, which has more than half the indicators that make up the SEVI which are classified as high or very high vulnerability. There are no cases of very low socio-environmental vulnerability in this planning area. The population of this region has been the object of several studies about its high social vulnerability ([66]).

PA 2 shows very different behavior from PA 1. 71.2% of the region is classified as having low socio-environmental vulnerability, 21.0% as having medium vulnerability and 7.8% as having high vulnerability. There are no cases of very low or very high socio-environmental vulnerability. The high point of the high-vulnerability region is the Rocinha *favela* with a population of 69,161, according to the 2010 Census, making it the largest *favela* in Brazil.

Rocinha has high or very high indicators of the socio-economic component, low indicators of urban infrastructure, very high susceptibility to slippage and high infant mortality. Although there have been investments in infrastructure, such as water supply, sewage and rubbish collection, other socio-economic problems concerning health and environmental risk are still very much present in this region.

Over half of the PA 3 region is characterized as having medium socio-environmental vulnerability (59.8%), 20.7% as having low vulnerability, 18.0% as having high and 1.5% very high socio-environmental vulnerability. In the region classified as having very high vulnerability, the Fazenda Botafogo and Bairro da Pedreira *favelas* are prominent. In these two regions all the socio-economic indicators, in addition to the infant mortality indicator, are classified as very high.

PA 4 is divided between low (43.3%) and medium (43.5%) socio-environmental vulnerability. The high-vulnerability areas occupy 13.2% of this region. The high point of the areas of high vulnerability lies in the Rio das Pedras *favela*, the third largest in Brazil, which according to the 2010 Census has 54,793 inhabitants. This region has practically all the indicators of the socio-economic component classified as high or very high vulnerability, very high risks of inundation and flooding, as well as very high violent lethality.

The PA 5 region is divided into areas of average (42.6%) and high (48.5%) socio-environmental vulnerability. Only 2.0% of this region is classified as having low vulnerability. PA 5 has the highest percentage of very high socio-environmental vulnerability in relation to other planning areas (6.9%), concentrated mainly in the neighbourhoods of Guaratiba, Paciência and Santa Cruz, which are not necessarily *favela* areas*.*

**FIGURE 5:**
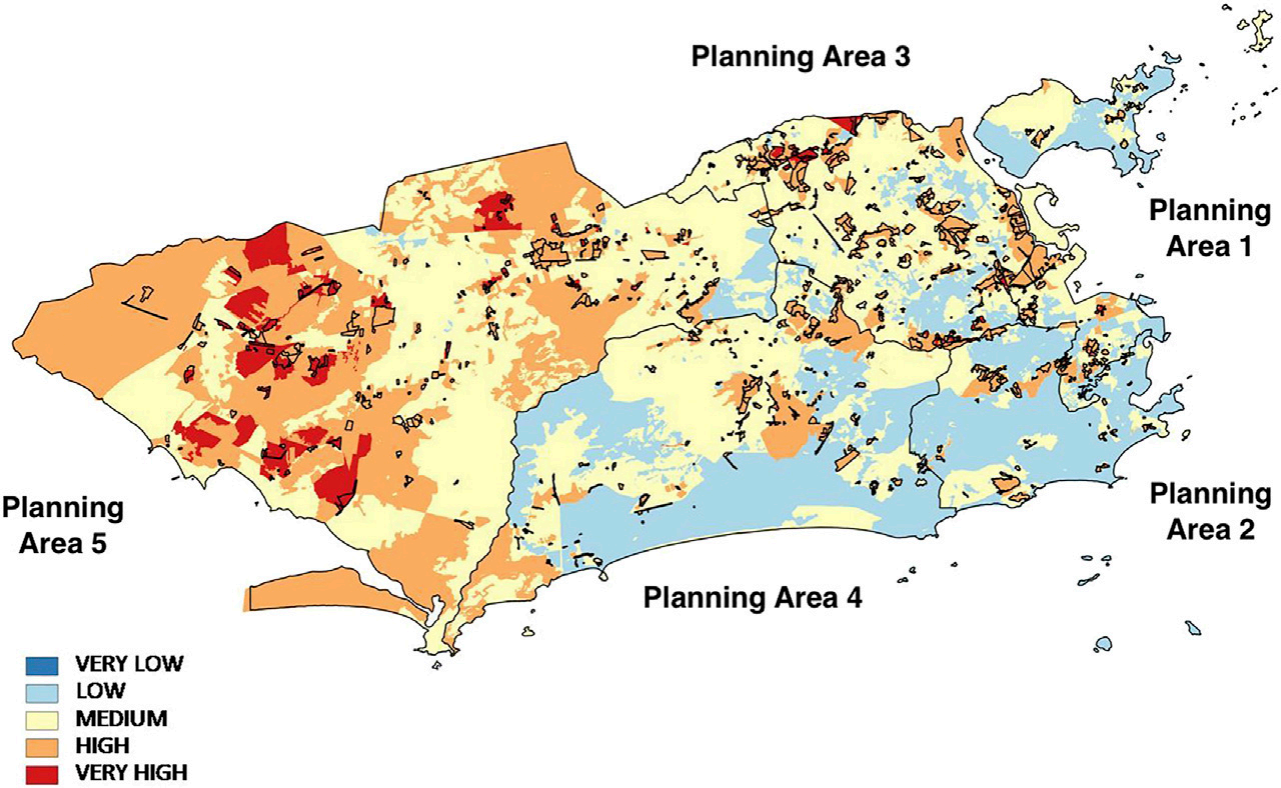
Map of the Social and Environmental Vulnerability Index and Subnormal Clusters in 2010. Source: Prepared by the authors.

When we analyze the final results of the SEVI for the entire municipality of Rio de Janeiro, it can be seen that the majority of the region is classified as having medium socio-environmental vulnerability (44.0%). Then 30.5% of the territory is classified as a having high socio-environmental vulnerability, a situation that is more prominent in PA 5.21.9% of the territory was classified as having low socio-environmental vulnerability, a situation prevailing in PAs 1, 2 and 4. 3.6% was classified as having very high socio-environmental vulnerability, with a higher concentration in PA 5. There were no cases of very low socio-environmental vulnerability (Figure 5).

## Discussion

According to Malczewski and Ogryczak [67], since the 1990s the use of the multi-criteria analysis methodology has been growing in territorial and urban planning. Currently, multi-criteria analysis is increasingly integrated into GISs, creating a robust tool to aid spatial analysis processes through modelling and to support decision-making in issues with spatial distribution and consequences. Efforts to integrate GIS and multi-criteria analysis in the late 1980s and early 1990s may be associated with increasing GIS development [61]. Sharifi et al. [68] also claim that the integration of GIS and multi-criteria analysis provides an important methodology in the creation of options to reduce environmental and socio-economic impacts, as well as to assess and solve these impacts in the territory.

The integration methodology between GIS and multi-criteria analysis used in the SEVI calculation corroborates other studies [69], where the author states that the integration between multi-criteria decision-making techniques and SIGs represents a considerable advance in spatial analysis involving urban planning, compared to the conventional map overlay approaches.

The importance of this methodology can be seen from the growing number of publications in the most diverse areas and regions of the world. Examples include the studies: where this methodology is used in land use planning in Switzerland [70]; when identifying sites for the construction of sanitary landfills in the Lake Beys region of Turkey [71]; in the construction of a flood risk map in Terengganu, Malaysia [72]; in locating potential sites for ecotourism in Kenya [73]; when identifying suitable regions for urban development in Ulaanbaatar, Mongolia [74]; in Brazil, in the analysis on the social fragility of the urbanized area of the Viçosa - MG municipality [75]; to determine the Sustainability Index of the Sugar-Ethanol Sector [76]; and in the analysis of the quality of urban life of the population in the city of Rio de Janeiro [77].

In this way, the multi-criteria analysis methodology integrated in GIS is being consolidated as an extremely useful resource in public and private management. For this purpose, it is necessary to understand the tool as a resource for reflecting on practices and an aid to decision-making, ensuring transparency and the possibility of incorporating subjective value judgments in the process [78]. In the specific case of this article, the integration of the methodologies aims to assist the processes of urban planning and land management.

Socio-environmental vulnerability is directly related to the urbanization of Brazil, where in 2010 over 80% of the Brazilian population lived in urban areas. In addition to concentrated urbanization, this change occurred in only a few decades, so the infrastructure of these cities did not keep pace with such growth. This rapid and disorderly process of urbanization resulted in a number of consequences, most of them negative. The lack of urban planning and of a less concentration-oriented economic policy contributed to the occurrence of some problems that persist to this day. One of the main problems arising from accelerated urbanization in Brazil was the concentration of wealth and consequently an increase in inequality [79].

This unequal formation of the social structure is expressed in the urban structure, i.e., the right to the city is not just and equal for all its inhabitants. Therefore, the most vulnerable groups suffer from socio-spatial segregation. In the case of the municipality of Rio de Janeiro this segregation occurs in *favela* regions, confirming Rodrigues [80] observations regarding the struggle for the right to the city.

From the results found in this article, it can be seen that the highest SEVI values are found in *favela* regions, thus confirming that the poorest populations suffer most of the negative effects of urbanization, confirming initial studies [7] where the authors discuss the variations of vulnerability in time and space among different social groups. The struggle for the right to the city and for the right to housing, one of its central components, emerged as a counterpoint to a model of exclusionary urbanization that over decades of accelerated urbanization absorbed large contingents of the poorest people in a few large cities, without ever effectively integrating them into the cities ([81–83]). In addition to the socio-economic problems, one of the dimensions of the struggle for the right to the city is the right to a healthy environment [84, 85], which requires access to sanitation, housing, security, infrastructure and health policies. The methodology and the results obtained for the SEVI can support these policies, contributing to the minimization of socio-spatial segregation and consequently to a change in the current model of urbanization so that all residents have the same right to the city.

### Conclusion

The methodology of integration between multi-criteria analysis and GIS developed in the construction of the SEVI represents an important tool for defining and validating policies for groups in situations of vulnerability. The creation of an index-map for the SEVI makes it easier to see the important aspects of the vulnerability processes, enabling the disaggregation of its components into maps, as well as maps of the indicators used in its construction. In this way it is possible to identify priority areas lacking specific policies and also to foster their monitoring.

The use of socio-economic, urban infrastructure, environmental, health and safety components in the construction of the SEVI is a combination that fully represents socio-environmental vulnerability and, in the specific case of this study, the reality of the most vulnerable groups in the municipality of Rio de Janeiro. These results should be taken into consideration by public authorities and other bodies dealing with this problematic context, so as to reduce situations of vulnerability and democratize the right to the city.

In order to apply it in other regions, we suggest the inclusion and/or substitution of indicators according to the reality of the region to be analyzed. In this way, through spatial knowledge of the most vulnerable areas it is possible to subsidize the creation of preparedness and response plans to deal with these problems and consequently to mitigate them.

## Data Availability

The datasets presented in this study can be found in online repositories. The names of the repository/repositories and accession number can be found below: <b>http://www.ppe.ufrj.br/index.php/pt/publicacoes/teses-e-dissertacoes/2018/183-vulnerabilidade-socioambiental-proposta-metodologica-e-diagnostico-para-o-municipio-do-rio-de-janeiro</b>.
